# Effects of Soluble Dietary Fibre on Exercise Performance and Perception of Fatigue in Young Basketball Players

**DOI:** 10.17113/ftb.61.03.23.8124

**Published:** 2023-09

**Authors:** Edin Hadžić, Antonio Starcevic, Tomislav Rupčić, Jurica Zucko, Toni Čvrljak, Ira Renko, Damir Knjaz, Dario Novak

**Affiliations:** 1 University of Zagreb, Faculty of Food Technology and Biotechnology, Pierottijeva 6, 10000 Zagreb, Croatia; 2 University of Zagreb, Faculty of Kinesiology, Horvaćanski zavoj 15, 10110 Zagreb, Croatia; 3 University of Zagreb, Faculty of Mining, Geology and Petroleum Engineering, Pierottijeva 6, 10000 Zagreb, Croatia

**Keywords:** exercise performance, dietary fibre, rating of perceived exertion (RPE), microbiome, amplicon sequencing

## Abstract

**Research background:**

In this study, we investigated the effects of soluble dietary fibre on improving neuromuscular and cardiovascular endurance and perception of fatigue in a closely monitored group of basketball players. Prebiotics have been sidelined in sports nutrition and their effect on performance remains poorly investigated and understood.

**Experimental approach:**

Eighteen healthy male basketball players were divided into two groups; one received 17 g/day of soluble dietary fibre (Nutriose®) for four weeks and the other group received placebo. Their morphological characteristics, neuromuscular and cardiovascular endurance, and rating of perceived exertion according to the rating of perceived exertion (RPE) scale were assessed. Measurements were taken before supplementation and after four weeks of supplementation. Faecal samples were collected from all participants immediately before and after the supplementation period, their total DNA extracted and sent for amplicon sequencing.

**Results and conclusions:**

In this study, fibre had no statistically significant effect on the vertical-type explosive power, no statistically significant effect on sprint-type explosive power, nor on aerobic and anaerobic endurance in the experimental group. Soluble fibre had a statistically significant effect on reducing the rating of perceived exertion of basketball players during the competitive part of the season (RPE 7.27±0.04 *versus* 8.82±0.81). This was confirmed by two-way ANOVA with replication, which showed that within-group interaction (p=0.0193), before and after dietary intake (p=0.0049), and between-group interaction before and after dietary intake (p=0.0313) had a significant effect on the result. The overall conclusion of the study is that soluble dietary fibre supplementation does not improve neuromuscular and cardiovascular endurance over a 4-week period. However, fibre supplementation could have a significant effect on reducing the rating of perceived exertion, as shown by the statistics. Both amplicon sequencing and subsequent bioinformatics results suggest that this could be the result of the beneficial effect on the intestinal microbiota and its metabolites.

**Novelty and scientific contribution:**

This work highlights the importance of prebiotics in sports nutrition. Dietary fibre has been a neglected component of sports nutrition. This study demonstrated a statistically significant positive effect on the perception of fatigue, highlighting the need for further studies in this direction.

## INTRODUCTION

In modern sport, nutrition is becoming an increasingly important factor on the path to achieving top results. Macro- and micronutrients already have a proven place in sports nutrition, and the effects of prebiotics on athletic performance are increasingly being observed (*1*). Prebiotics are both soluble and insoluble, indigestible fibre that stimulate the growth and activity of probiotics, *i.e*. intestinal microorganisms or microbiota (*2*, *3*). Dietary fibre is inaccessible to human digestive enzymes but available to the intestinal microbiota. As such, it affects the composition of the intestinal microbiota and its metabolites, and thus athletic performance (*4*).

To determine the effect of supplementation with prebiotic fibre on athletic performance, several criteria must be met: prebiotic potential, effect on changes in athlete morphology, fibre utilisation and the absence of negative side effects of supplementation (*5*, *6*). The prebiotic potential of soluble fibre has been shown in studies conducted predominantly on the general population, which showed that the intake of 10, 15 and 20 g/day of soluble fibre influences the composition of the intestinal microbiota towards proliferation of healthy and inhibition of pathogenic bacteria (*7*). Consumption of fibre for 14 days stimulated better growth of bacteria of the genus *Bacteroides* and inhibited the species *Clostridium perfringens*, the main cause of food poisoning. Studies show that intake of even 10 g/day for 8 weeks has a measurable effect on the hypothalamic-pituitary gland-adrenal gland axis and immune system in women with type 2 diabetes, improving mental health and immunity. A study conducted on 120 overweight Chinese men confirmed the beneficial effects of consuming up to 34 g/day of fibre in several smaller doses over a 12-week period on improving metabolic syndrome symptoms, alleviating insulin resistance and reducing body mass (*8*). A more detailed explanation for the effect of fibre on satiety and thus on body mass, body composition and energy intake is that fibre taken during breakfast reduces the concentration of ghrelin (a hormone that stimulates hunger) even at lunchtime, even after consuming a low-calorie breakfast (*9*).

A study of side effects conducted on 12 healthy men suggests that taking up to 50 g of fibre throughout the day over a 24- and 48-hour period does not cause any indigestion problems. The observed effects on bloating, gastrointestinal pain, vomiting, diarrhoea and stool consistency showed no differences between the control and experimental groups (*5*). A study of 43 subjects who consumed dietary fibre during 4 weeks, with total fibre content reaching 30–45 g/day, showed good tolerance and prebiotic function (*6*). However, in a study conducted on 10 subjects who consumed 10, 30 and 60 g/day for 7 days and another 10 who consumed 15, 45 and 80 g/day, bloating without diarrhoea or vomiting was observed when more than 60 and 80 g/day of fibre were consumed (*7*). The aim of the study conducted on 30 subjects was to determine the maximal tolerable amount of fermented soluble fibre (from wheat) without side effects. The study also determined the effect of the wheat dextrin by analysing the enzyme activity in the subjects’ faeces. The subjects consumed between 10 and 80 g/day of fibre for 7 days, and it was found that at the highest amount of fibre consumed, the residue in the colon increased to 13 %. As expected, the percentage depends on the consumed amount. A small amount of residue in faeces indicated that 87 % or more of the fibre is digested or fermented in the gastrointestinal tract (*9*).

Dietary supplements are increasingly used to help athletes achieve maximum athletic performance. Although there is insufficient data on the use of prebiotic supplements, a few studies indicate their positive effects (*4*). The main motivation behind this research is to evaluate these reported positive effects without compromising the results by adding too many new, sport-specific tests. The reason for this simpler approach is to avoid unnecessary testing in a relatively short period of time, as it could overwhelm participants with additional fatigue, which could negatively affect study outcomes. According to the available research showing the direct impact of training duration on the neuromuscular performance of the lower extremities, the objective is to evaluate the effect of controlled soluble fibre supplementation by relying on the already established neuromuscular performance tests used in basketball players (*10*). The study was conducted on 20 healthy university athletes who consumed a relatively low dose of 6 g/day of fibre over a period of 4 weeks. The results of this study indicate an improvement in aerobic and anaerobic capacity and different effects on stress biomarkers in saliva (*4*). This suggests that soluble fibre has the potential to alter the intestinal microbiota that uses them as food, resulting in more metabolites such as short-chain fatty acids with proven positive effects on the metabolism. In addition, they may also have an anti-stress effect and delay the perception of fatigue in athletes (*11*). Our hypothesis is that soluble dietary fibre supplementation will improve neuromuscular and cardiovascular fitness and the perception of fatigue. The primary objective of this study is to determine the impact of soluble dietary fibre on athletic performance optimisation (improving cardiovascular endurance and explosive power). The secondary objective is to determine the impact of soluble dietary fibre on delaying the perception of fatigue in basketball players during the competition period.

## PARTICIPANTS AND METHODS

### Participants

Eighteen healthy adult male professional basketball players from the same basketball team, average age (18.4±0.7) years, average height (193.6±6.4) cm and average mass (85.3±7.6) kg, participated in the study. Participants who had taken antibiotics in the last 3 months, participants with type 1 and type 2 diabetes, and participants who had taken other ergogenic agents or dietary supplements in the last 30 days before the start of the study were excluded. Participants were selected from a single basketball team without changing participants or adding separate training sessions during the competition season within the study period to eliminate bias. In addition, athletes received a fibre supplement during the competition season to limit the possibility of improvements in aerobic and anaerobic performance due to changes in training volume as the pre-competition period. All participants took part in both morning and afternoon training sessions. Morning training consisted of 60–90 min of conditioning in the gym (Monday, Tuesday, Thursday and Friday), while afternoon training consisted of teamwork on the field (Monday, Tuesday and Wednesday for 90–120 min and Thursday and Friday for 80–90 min).

Every Saturday there was a single league match and Sunday was a day off. If a participant had to skip a scheduled training session, this was to be replaced by an additional training session on a Sunday, with the same volume and load as the skipped training. The training load was higher early in the week and was gradually reduced before each Saturday match. As both the control and supplement groups had the same average mass/height, the chances for anthropometric parameters to influence the results were very limited at best. Since the Covid-19 regime was being enforced during the study period (from September 2021 until the end of October 2021), it was easier to monitor participants' off-field behaviour. No participant had to be excluded because of Covid-19 infection. Participants included seven former or current Croatian national team players in more than one age group and the head coach had an International Basketball Federation (FIBA) licence. Participants were advised not to change their dietary habits for two weeks before the study and during the study. The participants gave their written consent to take part in the study. They were randomly divided into two groups: the experimental group, which received the tested fibre, and the control group, which received the control. The research was approved by the Ethics Committee of the School of Medicine in Zagreb, class: 641-01/21-02/01. Using G*Power software (*12*) (v. 3.1.9.2; Heinrich Heine University Dusseldorf, Dusseldorf, Germany), it was estimated that 18 participants would represent an appropriate sample size to obtain an acceptable confidence level of 80 % for the interpretation of the results.

### Study design

This was a double-blind study in which an independent statistician was in charge of the randomisation of respondents into the two aforementioned groups. The study lasted a total of four weeks, during which both groups were continuously supplemented with fibre/control. Before the start of the supplementation treatment, morphological characteristics and tests to assess explosive power as well as aerobic and anaerobic capacities were measured first. After four weeks of fibre supplementation during the competitive part of the season, final measurements of morphological characteristics, tests assessing explosive power, aerobic and anaerobic capacities were repeated. After each training session or match, participants rated the intensity of their exercise (Rating of Perceived Exertion) on the RPE scale using the mobile phone messaging application (*13*).

### Morphological characteristics

Anthropometric measurements were performed twice – before and after dietary intake. Following variables were measured for the assessment of morphological characteristics: body height, body mass, upper arm skinfold, back skinfold, chest skinfold, abdomen skinfold, suprailiac skinfold, thigh skinfold, axillary skinfold and fat percentage calculated from seven skinfolds using an algorithm (*14*).

To estimate the body fat percentage, the general equation of body density (*ρ*) for men was used (*14*):



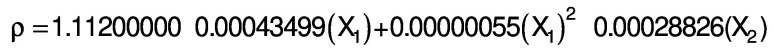



where *ρ* is the body density, X_1_ is the sum of seven skinfolds and X_2_ is the age in years. The determined body density (*ρ*) was used to estimate the body fat percentage with a Brozek formula (*15*):



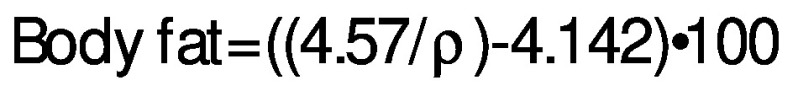



### Neuromuscular and cardiovascular fitness

To assess the athletic performance of athletes, emphasis was placed on the explosive power of the lower extremities (concentric and eccentric contractions). Following tests were used to assess sport specific performance: explosive power of the vertical type (squat jump (SJ), countermovement jump (CMJ), countermovement jump left foot (CMJLF) and right foot (CMJRF), maximum vertical jump (VJmax), repeated jumps 15 s (RJ15S) and five repeated stiffness jumps (RSJ5)) with Optojump system (Microgate, Bolzano, Italy) and the explosive power (EPO) of the sprint type (5 m sprint - EP05M, 10 m sprint - EP10M and 20 m sprint - EP20M) with Powertimer analyser (Newtest Oy, Oulu, Finland) (*16*). To assess cardiovascular endurance, aerobic endurance tests (Beep test) and anaerobic endurance tests 300MRT (300 metre test (15×20)) with Powertimer analyser (Newtest Oy) were used.

### Fibre supplementation

Nutriose® (Roquette Frères, Lestrem, France), which is derived from corn and used in the study, is generally well tolerated and no allergic reactions have been reported. This soluble fibre (per 10 g) contains <0.3 % protein, 0 g fat, <15 % mono- and disaccharides, <0.5 % residue on ignition (sulphated ash) and 8.5 g dextrin. Participants were informed about this in the informed consent form. The experimental group (*N*=9) consumed a total of 20 g of this fibre-containing product, divided into two separate 10-gramme doses daily, for four weeks, and the control group (*N*=9) consumed a control carbohydrate in the form of maltodextrin (Roquette Frères) in completely the same way and packaging. Maltodextrin has the same consistency as Nutriose®, similar solubility and taste as Nutriose®, and although some recent studies report that it may also affect gut microbiota (*17*), it remains one of the most widely used placebos or control compounds when investigating fibre prebiotic effects (*18*). Participants took both supplements after each training session (two training sessions per day) in the presence of the principal investigator, who was not informed about the contents of the supplement. On a morning off (Wednesday) or a day off (Sunday), participants were instructed to consume the supplement without supervision and to send pictures *via* mobile phone messaging application of the empty sachet containing either the fibre supplement or the control that had been given to them the previous day.

The duration of the supplement intake was determined according to a study that investigated the effect of fibre on athletic performance under the premise of the Covid-19 pandemic, which had imposed some restrictions (4). These restrictions had affected the so-called friendly competitions, which haven't been scheduled since they increase the inevitable risk of participants having to undergo isolation if they came into contact with someone infected. Even with the imposed restrictions, longer study duration would inevitably increase the risks of participant dropout. All things considered, 4 weeks of supplementation has been selected as an acceptable compromise, although longer study duration could arguably provide additional feedback.

### Recording of the rating of perceived exertion scale

To understand the effect of fibre consumption on the perception of fatigue, the participants recorded a rating of perceived exertion after each training session or match and sent it *via* a mobile messaging application. Perceptions of fatigue referred to the participant's perception during a particular training session or match, rather than the participant’s state after the training session or match itself. The RPE scale is a tool for monitoring perceived response to training, as a method of determining physical exertion during exercise (*19*). The Borg’s CR10 had a scale from 0 to 10 (rating of perceived exertion (RPE)), and the original RPE scale, developed more than 40 years ago, was primarily used to monitor aerobic load, but is now widely used to monitor athletes (*20*).

### Microbiome analysis

Faecal samples were collected from the participants in a sterile container and immediately frozen at -20 °C. On the same day, participants brought the frozen samples to training, from where they were transferred to the -80 °C freezer using a portable freezer box. The faecal samples were stored at -80 °C until further analysis. DNA was extracted from 0.5 g of thawed faecal matter using the QIAamp® PowerFecal® DNA kit (Qiagen, Hilden, Germany) according to the manufacturer's instructions. DNA concentration was quantified using the Quantus™ Fluorometer (Promega, Leiden, The Netherlands) and sent to the Molecular Research (MRDNA) Lab (Shallowater, TX, USA) for amplicon sequencing of variable regions 3 and 4 of the 16S rRNA gene with the primer set 341F (5’-CCTAYGGGRBGCASCAG-3’) and 806R (5’-GGACTACNNGGGTATCTAAT-3’). The raw sequence data were downloaded from Illumina BaseSpace Sequence Hub in the form of paired-end, multiplexed fastq files. The files were first demultiplexed using sabre (*21*) (https://github.com/najoshi/sabre) and imported into the 'Quantitative Insights Into Microbial Ecology 2' (QIIME2) software, v. 2020.6 (*22*) *via* a manifest file. The imported sequences were quality filtered, trimmed, denoised, merged, screened for chimeras and used to create amplicon sequence variants (ASVs) using the DADA2 plugin (*23*). ASVs were assigned taxonomy using a pre-trained Naïve Bayes classifier. The classifier was trained on the SILVA version 138 database of reference sequences clustered at 99 % sequence similarity (*24*) using the QIIME2 feature-classifier plugin (*25*). The phylogenetic tree was generated using fasttree2 based on the MAFFT alignment of the ASVs as implemented in the q2-phylogeny plugin. The diversity and richness of all samples were estimated using alpha (observed features, Shannon’s index and Faith’s phylogenetic diversity) and beta (weighted and unweighted Unifrac) diversity metrics using the QIIME diversity plugin, subsampled at 10 000 reads per sample.

### Statistical analysis

The research data were analysed using conventional methods of descriptive statistics: arithmetic mean and standard deviation (S.D.). ANOVA 2×2 was used to analyse the variables between the initial and final measurements both within each group and between groups (Supplementary_ANOVA.xlsx, https:/doi.org/10.17632/3s432m9gyx.1). Statistica v. 13.3.0 software (*26*) was used for all analyses. Statistical comparisons were performed with commercially available software Prism v. 8.0 (*27*) and rate of perceived exertion (RPE) variables were analysed using a two-way repeated-measures analysis of variance (ANOVA) to evaluate the differences between trials. Tests of normality and assumptions were conducted, and if a significant violation was found, an appropriate adjustment to the degrees of freedom was made. For the time-to-exhaustion (TTE), the last time point was the subjective time to task failure. Two-tailed paired samples *t*-test was used to assess the differences between pre- and post-TTE in the cardiovascular assessments. Statistical significance was declared when p<0.05. Data are presented in the text and tables as mean±S.D. and in the figures as mean±SEM, unless otherwise stated.

Statistical significance of differences in microbial abundance was assessed using STAMP v. 2.1.3 software (*28*). Taxonomic assignments at species level obtained with QIIME2 were exported as a .csv file and prepared in the format required for STAMP. Statistical differences in microbiota abundance between the control and experimental groups were determined using a two-sided Welch’s t-test.

## RESULTS AND DISCUSSION

### Characteristics of the participants

Eighteen young, healthy adult basketball players, divided into two groups, met all the set criteria for participation and underwent all necessary measurements and tests from the beginning to the end of the study. The characteristics of the participants in the experimental (fibre supplement) and the control (control supplement) group are shown in Table 1.

**Table 1:** Characteristics of the participants

Variable	Initial		Final	
Experimental group	Control group	Experimental group	Control group
*m*(body)/kg	87.7±9.3	82.9±5.0	88.1±9.9	82.3±6.0
*w*(fat)/%	8.9±2.6	9.0±2.3	8.8±2.4	8.8±2.2
BMI	22.9±2.2	22.2±1.9	23.1±2.4	22.5±2.1

Results are presented as mean value±standard deviation (S.D.). BMI=body mass index

### Effects of Nutriose® on morphology

Both before and after the intake of the supplement, body mass (BM) was slightly higher in the experimental group than in the control group: (87.7±9.3) *versus* (82.9±5.0) kg and (88.1±9.9) *versus* (82.3±6.0) kg, respectively. The experimental and control groups had a similar percentage of body fat at the initial measurement (8.9±2.6) *versus* (9.0±2.3) %. After 4 weeks of training, the body fat measurements and the two-way ANOVA with replication showed that the group had no effect on the measured variable (p=0.945) in terms of body composition. There was no significant difference before and after (p=0.894) the intake of the supplement within each group and there was no significant interaction between groups before and after intake (p=0.979). The 3-day dietary logs were used to record the participants’ diet before the start of the supplementation. During the supplementation period, 24-hour recall was used once a week, previously unannounced in order to monitor participants’ diet. This was done specifically to determine total fibre intake from the regular diet both before and during the fibre supplementation, as shown in Table 2 below.

**Table 2:** Assessment of total dietary fibre intake: (1) based on 3-day dietary logs before supplementation, (2) based on four weekly 24-hour dietary recalls during fibre supplementation period, and (3) during the fibre supplementation period with supplementation included (Nutriose® or control in the form of maltodextrin)

Group	Total fibre (1)	Total fibre (2)	Total fibre+Nutriose® (3)
Experimental	28.4±11.8	29.6±11.2	46.6±11.2
Control	24.0±8.4	22.4±8.2	22.4±8.2

Results are presented as mean value±S,D.

The average fibre intake obtained through diet both before starting the supplement intake and during the intake showed a slight difference between the groups, but this was not significant. The dietary fibre intake from the regular diet was within a normal range in both the control and experimental groups, comparable to that observed in other EU countries. With supplementation, the total fibre intake of the experimental group increased to the recommended dose or slightly above, while the total fibre intake of the control group remained within the range of the normal Western diet (*29*).

### Effects of Nutriose® on neuromuscular fitness - Explosive power of vertical type

The results of explosive power of vertical type in tests SJ, CMJ and VJmax did not show statistical significance neither in the experimental group nor in the control group: SJ-Nutriose® (35.6±5.1) *versus* (39.3±5.8) cm and SJ-control (38.0±5.2) *versus* (40.0±3.8) cm (p=0.978, F=0.001 with F_crit_=4.149) within each group, before and after the intake (p=0.424, F=0.655 with F_crit_=4.149), and interaction between groups before and after the intake (p=0.713, F=0.138 with F_crit_=4.149), CMJ-Nutriose (40.52±5.99) *versus* (40.72±5.99) cm, CMJ-control (39.98±5.05) *versus* (40.78±3.91) cm (p=0.891, F=0.019 with F_crit_=4.149) within each group, before and after dietary intake (p=0.779, F=0.079 with F_crit_=4.149), and interaction between groups before and after (p=0.866, F=0.029 with F_crit_=4.149), VJmax-Nutriose (47.89±6.62) *versus* (48.18±6.16) cm, VJmax-control (46.69±4.99) *versus* (48.12±5.9) cm (p=0.754, F=0.100 with F_crit_=4.149) within each group, before and after the intervention (p=0.667, F=0.188 with F_crit_=4.149), and interaction between groups before and after (p=0.774, F=0.083 with F_crit_=4.149).

**Fig. 1:**
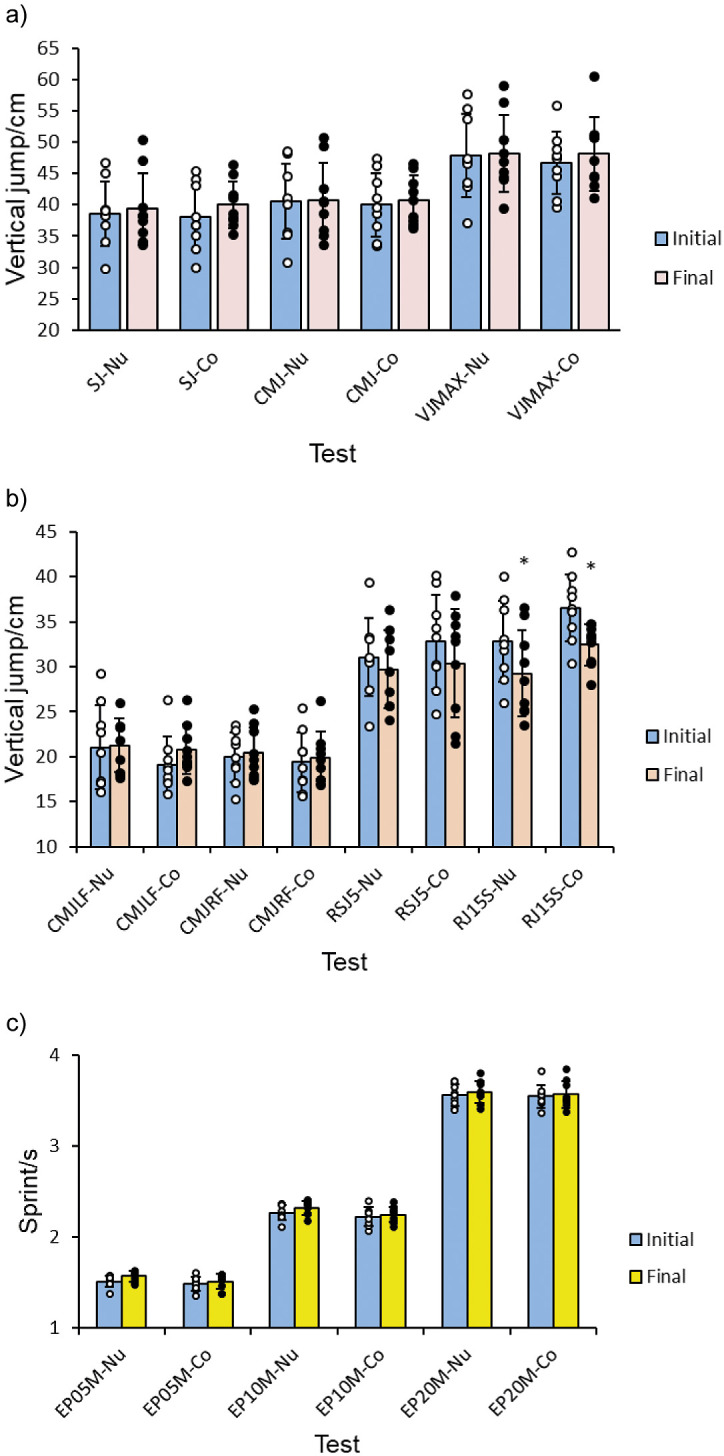
The effect of Nutriose (Nu) on: a) explosive power of vertical type, 1st set of tests. Values are shown as individual data and mean±S.D., b) explosive power of vertical type, 2nd set of tests. Values are shown as individual data and mean±S.D., and c) explosive power of sprint type. Values are shown as individual data and mean±S.D. Co=control, Nu=nutriose, SJ=squat jump, CMJ=countermovement jump, VJMAX=maximum vertical jump, CMJLF=countermovement jump left foot, CMJRF=countermovement jump right foot, RJ15S=repeated jumps 15 s, RSJ5=five repeated stiffness jumps, EP05M, EP10M and EP20M=explosive power 5, 10 and 20 metres, respectively

The results of explosive power of vertical type in tests CMJLF, CMJRF and RSJ5 also did not show statistical significance neither in the experimental group nor in the control group: CMJLF-Nutriose (21.04±4.66) *versus* (21.23±2.98) cm, CMJLF-control (19.12±3.07) *versus* (20.81±2.74) cm (p=0.315, F=1.041 with F_crit_=4.149) within each group, before and after dietary intake (p=0.419, F=0.668 with F_crit_=4.149), and interaction between groups before and after (p=0.518, F=0.426 with F_crit_=4.149), CMJRF-Nutriose (19.93±2.74) *versus* (20.41±2.87) cm, CMJRF-control (19.39±3.32) *versus* (19.87±2.88) (p=0.585, F=0.304 with F_crit_=4.149) within each group, before and after the intervention (p=0.234, F=2.054 with F_crit_=4.149), and interaction between groups before and after (p=1.0, F=0.001 with F_crit_=4.149), RSJ5-Nutriose (31.07±4.34) *versus* (29.68±4.34) cm, RSJ5-control (32.77±5.21) *versus* (30.39±6.01) cm (p=0.477, F=0.518 with F_crit_=4.149) within each group, before and after the intervention (p=0.269, F=1.265 with F_crit_=4.149), and interaction between groups before and after (p=0.769, F=0.087 with F_crit_=4.149). Results within groups RJ15S-Nutriose (32.8±4.5) *versus* (29.3±4.8) cm (p=0.013, F=6.895 with F_crit_=4.149), and RJ15S-control (36.6±3.7) *versus* (32.4±2.3) cm (p=0.0065, F=8.481 with F_crit_=4.149) showed statistical significance within the groups before and after the dietary intake. Interaction between groups before and after the intake of the supplement has had no significant impact on the result (p=0.829, F=0.047 with F_crit_=4.149), and therefore it was deemed not relevant. For clarity, the first three tests (SJ, CMJ, VJmax) are shown in Fig. 1a, and the other four tests are shown in Fig. 1b.

### Effects of Nutriose® on neuromuscular fitness - Explosive power of speed type

For explosive power of speed type (EP05M, EP10M and EP20M), the results within the experimental and control groups during the dietary intervention and the final results between groups did not show statistical significance. Results of the first test did not show statistical significance: EP05M-Nutriose (1.51±0.06) *versus* (1.57±0.06) s, EP05M-control (1.49±0.08) *versus* (1.51±0.08) s, within the groups (p=0.087, F=0.016 with F_crit_=4.149), before and after dietary intake (p=0.095, F=2.951 with F_crit_=4.149), and interaction between groups before and after dietary intake (p=0.471, F=0.532 with F_crit_=4.149). Also the other two tests did not show statistical significance: EP10M-Nutriose (2.27±0.08) *versus* (2.32±0.08) s, EP10M-control (3.2±0.1) *versus* (2.25±0.08) s, within the groups (p=0.062, F=3.750 with F_crit_=4.149), before and after dietary intake (p=0.204, F=1.683 with F_crit_=4.149), and interaction between groups before and after (p=0.589, F=0.297 with F_crit_=4.149), EP20M-Nutriose (3.6±0.1) *versus* (3.6±0.1) s and EP20M-control (3.6±0.1) *versus* (3.6±0.2) s, within the groups (p=0.697, F=0.0.155 with F_crit_=4.149), before and after the intervention (p=0.555, F=0.355 with F_crit_=4.149), and interaction between groups before and after (p=0.870, F=0.027 with F_crit_=4.149), which can be seen in Fig. 1c.

### Effects of Nutriose® on cardiovascular fitness

**Fig. 2:**
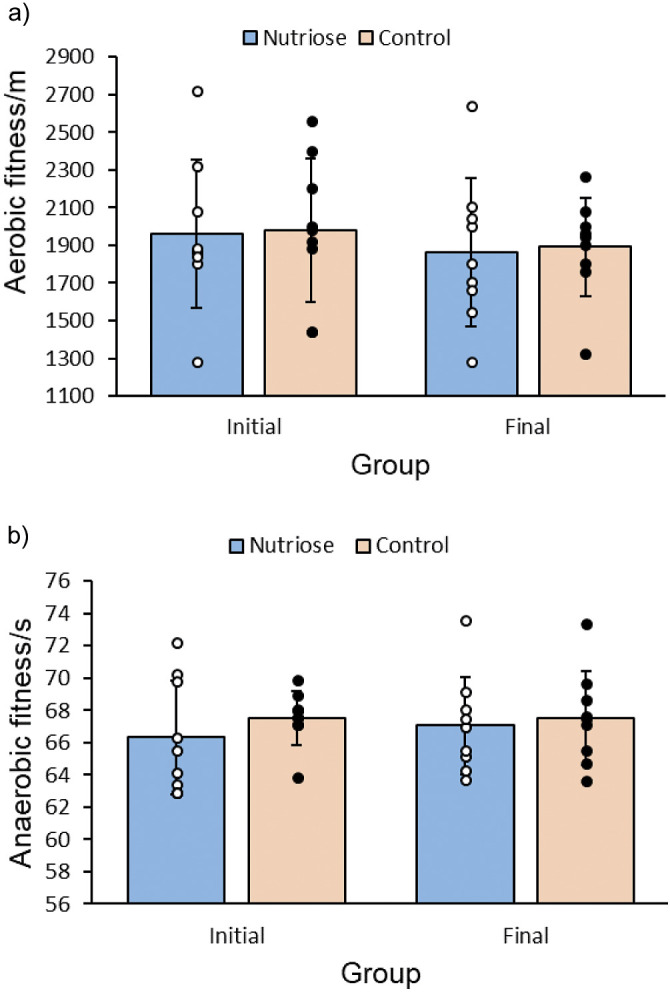
Effect of Nutriose® on: a) aerobic fitness, BEEP. Values are shown as individual data and mean±S.D., and b) anaerobic fitness, 300MRT. Values are shown as individual data and mean±S.D. BEEP=sound signal, MRT=metre run test

The results of the BEEP test in the experimental and control groups show similar values at the beginning ((1960.0±395) *versus* (1980±380) m) and at the end of the intervention ((1862±392) *versus* (1891±260) m). Four weeks of training showed that the group had no significant impact on the result (p=0.840, F=0.041 with F_crit_=4.149). Also, there was no significant difference in the results before and after the intake of dietary supplement (p=0.444, F=0.601 with F_crit_=4.149). There was also no significant interaction between groups before and after the intake (p=0.971, F=0.001 with F_crit_=4.149), as shown in Fig. 2a.

The 300MRT results show similar values at the beginning (experimental *versus* control: (66.3±3.5) *versus* (67.1±3.0)) and at the end of the intake (experimental *versus* control: (67.5±1.6) *versus* (67.5±2.9)). After four weeks of training, it was found that the group had no significant impact on the result (p=0.407, F=0.707 with F_crit_=4.149). Also, there was no noticeable difference in the results before and after the intake (p=0.721, F=0.129 with F_crit_=4.149). There was also no significant interaction between groups before and after the intake (p=0.695, F=0.157 with F_crit_=4.149). Final measurements also did not yield statistical significance between the groups of participants receiving fibre and the control ((67.1±3.0) *versus* (67.5±2.9)), as shown in Fig. 2b.

### Effects of Nutriose® on rating of perceived exertion

The values of RPE differ within the experimental group before and after supplement intake ((9.0±1.1) *versus* (7.3±0.4)) and at the end between the experimental and control groups (8.8±0.1 *versus* 7.3±0.4). Two-way ANOVA with replication showed that the group had a significant effect on the result (p=0.0193, F=6.472 with F_crit_=4.351). There was also a significant difference between the results before and after Nutriose supplement intake for RPE (p=0.0049, F=10.019 with F_crit_=4.351). There was also a significant interaction between groups, before and after Nutriose supplement intake (p=0.0313, F=5.3607 with F_crit_=4.351).

### Effects of Nutriose® on microbiome

**Fig. 3:**
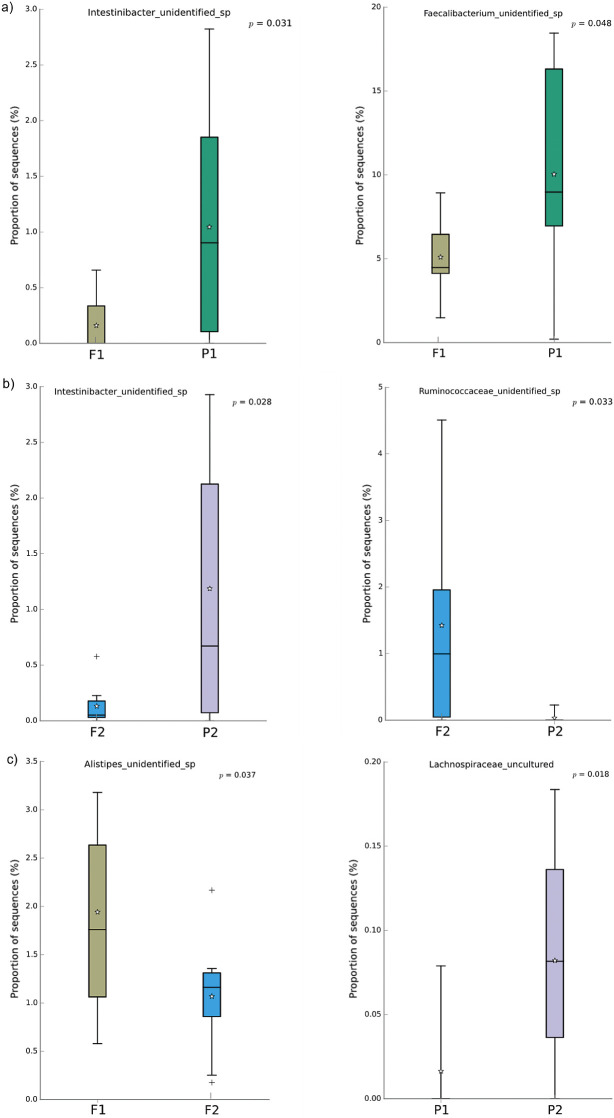
The impact of fibre supplementation on gut microbiome. Difference in the abundance of: a) an unidentified *Intestibacter* sp., b) an unidentified *Faecalibacterium* sp., c) an unidentified species from the genus *Intestinibacter,* d) an unidentified species from the genus *Ruminococcus*, e) an unidentified species from the genus *Alistipes* and f) the family Lachnospiraceae species. F1=fibre supplemented group before starting the intervention and F2=group supplemented with fibre after four weeks of the intervention, P1=group supplemented with placebo before starting the intervention and P2=group supplemented with placebo after four weeks of the intervention

The effect of fibre supplementation on the gut microbiome was assessed by monitoring relative taxonomic abundance (Fig. S1 and Fig. S2 at: https://doi.org/10.17632/3s432m9gyx.1) in both groups of participants at two time points: time point 1, immediately before starting supplementation intervention, and time point 2, immediately after 4 weeks of fibre supplementation. Both the control group, supplemented with maltodextrin before (P1) and 4 weeks after (P2) and Nutriose® fibre supplemented group before (F1) and 4 weeks after (F2) showed a similar distribution of microbial taxa (Suppl. Dataset 1, https://doi.org/10.17632/3s432m9gyx.1) at phylum level – with the phylum Firmicutes being the most abundant in both groups (61.1–69.2 %), followed by the phyla Bacteroidetes (22.3–28.9 %) and *Actinobacteria* (7.3–11.2 %). No significant differences in abundance were found on phylum level, both between groups and between different time points. On the family level, Lachnospiraceae was the most dominant in both groups, with abundance ranging from 21.2 to 29.5 %, and was more abundant in the group supplemented with Nutriose® fibre. The second most abundant family in both groups was Ruminococcaceae (17.9–18.8 %), followed by the Bacteroidaceae (8.5–17.5 %) and Bifidobacteriaceae (6.7–10.2 %) families. No significant differences in abundance were found at the family level. Only at the species level were significant differences in abundance found. The abundance of two species differed significantly between the control and the Nutriose® fibre supplemented groups at the time point 1 (P1 and F1) – an unidentified species from the genus *Intestinibacter* (p=0.031) and an unidentified species from the genus *Faecalibacterium* (p=0.048), both more abundant in the control group (P1) (Fig. 3a). One species – an unidentified species from the genus *Alistipes* showed a significant difference in relative abundance (p=0.037) between the two time points in the Nutriose® fibre supplemented group (F1 and F2) and showed a decrease in abundance during the test period of 4 weeks (Fig. 3c). In the group supplemented with the control in the form of maltodextrin, there were no species with significantly different abundance between the two time points when the faecal samples were taken. At the end of the treatment, two species differed significantly in relative abundance between the control group and the group receiving Nutriose® fibre – an unidentified species from the genus *Intestinibacter* (p=0.028), which was more abundant in the group taking maltodextrin, and an unidentified species from the genus *Ruminococcaceae* (p=0.033), which was more abundant in the group receiving Nutriose® fibre, as shown in Fig. 3b.

### General effects recorded

The aim of this study was to determine the effects of soluble dietary fibre intake on athletic performance. This study also examined the effect of soluble fibre on the anger-hostility score (negative mood), a test that assesses the effects on aggressive behaviour and negative emotions, but not on the rating of perceived exertion (RPE) (*30*). Although the above-mentioned study suggests an association between the consumption of soluble fibre and improvements in athletic performance in terms of aerobic and anaerobic capacity, we did not observe such improvements in our study. Our hypothesis was that 4 weeks of continuous fibre supplementation would have an observable effect on neuromuscular performance of lower extremities. This hypothesis was not confirmed by the results, as no significant effects were observed during this period. The second hypothesis was that this period of supplementation would affect both aerobic and anaerobic capacity of the participants, which was also not confirmed by the results. We measured the effect of soluble fibre supplementation on the neuromuscular performance of the lower extremities, *i.e*. the explosive power of the vertical type and sprint type, as well as on the rating of perceived exertion according to the RPE scale. Regarding body fat, young active basketball players with an already low percentage of body fat are not the ideal group to study the effect of fibre supplementation on its reduction (*31*). We were aware of this and considered the possibility of fibre affecting body fat composition, but we did not formulate it as a working hypothesis. It is well-known that without large-scale dietary intervention or more drastic changes in the volume and/or intensity of training, it is difficult to reduce the body fat percentage of athletes who already have less than 9 % body fat. However, the results of initial motor skill tests indicated some potential for improvement, which is consistent with previous studies (*32*).

The effect of training leads to changes in the composition of the intestinal microbiota, the bacteria that inhabit our digestive system, and some research data indicate that there is a difference in the composition of the intestinal microbiota between athletes and non-athletes (*33*). However, there are still no conclusive studies linking the consumption of probiotics or prebiotics to improved athletic performance (*34*). The majority of existing studies generally suggest the health benefits of consuming probiotics and prebiotics, but there is no strong evidence of their ergogenic properties (*33*, *34*).

The effects of soluble fibre on morphological characteristics, body mass and percentage of subcutaneous adipose tissue in overweight and obese individuals are well documented (*35*). In these cases, increased consumption of dietary fibre affects the reduction of adipose tissue, which is not the case in athletes in the competition program (*36*). Regarding fibre, the recommendations for athletes do not differ from those for the rest of the population, and the research results do not show significant effects on the increase of muscle mass or reducing subcutaneous adipose tissue. Accordingly, our study did not show a significant reduction of subcutaneous adipose tissue in participants whose percentage of subcutaneous adipose tissue was already below 9 %.

### Effects on neuromuscular fitness

Soluble fibre has a low and sometimes negative energy value. The antinutrient effects and the expected mass and adipose tissue loss are based on the properties of inducing satiety and affecting macronutrient absorption. The explosive power of the vertical type and sprint type depends on both body mass and fast-twitch muscle fibres. Because of the reduction of adipose tissue and thus body mass during training, the efficiency of explosive power of the vertical type may increase, and thus the height of the jump (*37*). In our study, we did not find a significant reduction in body mass and body fat, nor did we record statistically significant changes in jump height in seven different jump tests. RJ15S, a test of repetitive countermovement jumps, showed a statistically significant reduction in the jump height within the groups, but the final result between the groups had no statistical significance. The assumption is that the training process varies during the competition season and the total load is adjusted for the competition. We did not find any changes in the explosive power of speed type in this study. It is possible that either the dose or duration of soluble fibre supplementation used in our study was not sufficient to have a significant effect on adipose tissue loss as well as on fast-twitch muscle fibres.

### Effects on cardiovascular fitness

Research on the effect of probiotics on athletic performance is mainly focused on cardiovascular endurance, especially in endurance sports. The health benefits of probiotics and prebiotics in athletes may improve immunity by reducing oxidative stress and decrease respiratory tract infections, which is indirectly associated with a positive effect on athletic performance (*38*). With heavy physical exertion, such as endurance training, these positive effects could lead to a shorter period of recovery from infection and accelerated respiratory tract infection rehabilitation, leading to earlier return to training. Apart from the mentioned benefits, studies have not reported any ergogenic effects following consumption (*33*). There is a lack of research that would suggest a direct effect of prebiotics and probiotics on cardiovascular endurance (*4*). Prebiotic soluble fibre could play a role in an athlete’s organism on which their putative ergogenic effect could be based, by increasing the feeling of satiety, promoting a healthy microbiota and its useful metabolites, such as enabling the synthesis of short–chain fatty acids (SCFA) (*2*). The synthesis of SCFA could explain the prolonged energy intake and improved anaerobic capacity. However, in our study we did not find a statistically significant effect on cardiovascular endurance, *i.e*. on aerobic and anaerobic capacity. This suggests that the dose of soluble fibre as well as the duration of the intervention may not have been sufficient for a significant effect on the cardiovascular fitness of the athletes.

### Effects on rating of perceived exertion

One of the additional models used to assess the limiting factors of submaximal training is the psychological model. Physiological, metabolic and biomechanical factors may be the cause of the limitations of this model (*39*). Soluble fibre can produce various effects through its role in host and microbiota metabolism by stimulating the growth of healthy microbiota and the production of its metabolites, such as SCFA, which is mainly metabolised in intestinal and hepatic enterocytes and can be found in plasma, pancreas and brain (*40*).

SCFAs can also act as signalling molecules that modulate the metabolism of other substrates (*40*). The only study measuring the concentration of SCFAs in the human faeces shows that athletes have a higher concentration of SCFAs in their faeces than the control group, which consists of the general population (*41*). This could indicate a greater microbiota diversity and/or positive effect of training on microbiota composition. It is known that SCFAs formed as a product of the intestinal microbiota are used directly in the striated skeletal muscles and that there are receptors for SCFAs in the entire nervous system that may enhance sympathetic activity (*33*). These studies raise questions about the possible effect of soluble fibre as a substrate for SCFA production on athletic performance.

The effect of soluble fibre on the RPE of athletes has not been extensively researched (*2*, *42*). Our study showed statistical significance in reducing the rating of perceived exertion within the experimental group from the start of supplementation to the end and in the final results between the experimental and control groups. Thus, consumption of Nutriose® soluble fibre for 4 weeks, 2×10 g/day (containing 17 g fibre in total) reduced the rating of perceived exertion of the participants in the experimental group receiving fibre. The explanation for these results could be the aforementioned role of SCFA, but it would be interesting to measure the concentration of SCFA in the blood plasma on training and competition, during the entire dietary intervention. Although training has a positive effect on the health of the intestinal microbiota, the lack of research in humans raises the question of whether the health of the intestinal microbiota and prebiotics have an effect on athletic performance. More research is needed to answer this question more conclusively. The shortcomings of our study can be found in the generalised amount of soluble fibre supplemented daily (17 g/day), which could ultimately be lower than the dose needed by individual microbiota, given the fact that western diet is known to lack fibre (*43*). Also, limitations of the study can be found in a small sample of 18 basketball players of only one gender, individual diet during the experiment and the 4-week period was relatively short, but due to the competition season and the specifics of the training period, the participants were able to meet all the requirements of the research follow-up so a compromise had to be made.

### Effects on gut microbiome

While gut microbiota is associated with numerous conditions, its impact on athletic performance is largely underexplored. Terms and concepts such as dysbiosis, probiotics, prebiotics, healthy microbiota or healthy bacteria are increasingly used; however, the existing definitions for these terms are vague at best (*44*). In this study, a latent study theme was the prebiotic potential of adding more soluble fibre to existing diet, without introducing any other dietary changes. Although the time period in our study was quite short and the number of participants was limited, these limitations also have some advantages. The notable benefit being the possibility to study a highly homogenous and strictly controlled group of participants in a time frame in which any significant difference recorded is likely to result from the studied dietary intervention. To assess the impact of the added fibre on gut microbiome, faecal samples were taken immediately before the fibre supplementation and immediately after the four weeks of supplementation. These two time points provided an insight into the composition of gut microbiota in form of relative abundance of its constituent bacterial species. In the control group receiving maltodextrin, there were no recorded species with significantly different abundance between the two time points when the faecal samples were taken. However, there were differences between the control and group receiving dietary fibre in this time period. One notable difference was the relative abundance of bacteria belonging to the Lachnospiraceae family, which is one of the core families of the human gut microbiota known to play a role in both health and disease (*45*). Members of the Lachnospiraceae taxa are involved in the production of various compounds that affect human health, primarily SCFA were more abundant in the fibre receiving group. There is evidence that adding more fibre to a diet can lead to more SCFA-producing bacteria in the gut, which can even affect our behaviour (*46*). The genera *Intestinibacter* and *Ruminococcus* also showed a notable difference between the groups, with fewer *Intestinibacter* and more *Ruminococcus* in the fibre receiving group at the end of the experiment, compared to control. These bacteria are most likely among the fastest responders to the added fibre to our diet, since the dietary fibre consumption for even shorter time confirms these results (*47*). Finally, *Alistipes*, isolated primarily from clinical samples, is a relatively new addition to the microbiota although much less abundant than other genus members of the phylum Bacteroidetes, which are often associated with conditions of dysbiosis and disease (*48*). The relative abundance of these bacteria decreased significantly in the group receiving Nutriose®. The fact that these bacteria are associated with a number of dysbiosis-related health issues indicates prebiotic potential of adding soluble fibre to the diet of athletes (*49*).

## CONCLUSIONS

The results of this study suggest that soluble dietary fibre does not improve neuromuscular performance or cardiovascular endurance in the short term. However, it can be concluded at the same time that fibre supplementation influenced the rating of perceived exertion, which could be the indirect result of the positive impact of soluble fibre on the intestinal microbiota and its metabolites. Since there were no reported side effects during the course of the study, this could lead to a conclusion that a moderate increase in fibre intake in athletes has the potential to support the intestinal microbiota and, conversely, positively impact training-induced perception of fatigue. Further research is needed to determine whether intestinal microbiota health and prebiotics have an effect on athletic performance, which depends on different doses or duration of supplementation.

## Data Availability

All supplementary materials are available at Mendeley Data, published under CC BY 4.0, where they can be accessed freely: Starcevic, Antonio; Hadzic, Edin (2023), “Effects of soluble dietary fiber on Exercise Performance and Perception of Fatigue in Young Basketball Players”, Mendeley Data, V1, https://doi.org/10.17632/3s432m9gyx.1. The entire study has also been published in the ClinicalTrials.gov Identifier: NCT05726435, under the title: ’University of Zagreb Protocol Record IP-2016-06-3509, Effects of Soluble Dietary Fiber on Sport Efficiency and Fatigue Delay in Top Basketball Players’'.
